# Outcomes of Sutureless Iris-Claw Lens Implantation

**DOI:** 10.1155/2016/7013709

**Published:** 2016-08-24

**Authors:** Tomasz Choragiewicz, Robert Rejdak, Andrzej Grzybowski, Katarzyna Nowomiejska, Joanna Moneta-Wielgoś, Małgorzata Ozimek, Anselm G. M. Jünemann

**Affiliations:** ^1^Department of General Ophthalmology, Medical University of Lublin, Ulica Chmielna 1, 20-079 Lublin, Poland; ^2^Department of Didactics and Medical Simulation, Human Anatomy Chair, Medical University of Lublin, Ulica Jaczewskiego 4, 20-090 Lublin, Poland; ^3^Department of Ophthalmology, Poznań City Hospital, Ulica Szwajcarska 3, 61-285 Poznan, Poland; ^4^Chair of Ophthalmology, University of Warmia and Mazury, Ulica Warszawska 30, 10-082 Olsztyn, Poland; ^5^Department of Ophthalmology, University Eye Hospital, Rostock, Germany

## Abstract

*Purpose*. To evaluate the indications, refraction, and visual and safety outcomes of iris-claw intraocular lens implanted retropupillary with sutureless technique during primary or secondary operation.* Methods*. Retrospective study of case series. The Haigis formula was used to calculate intraocular lens power. In all cases the wound was closed without suturing.* Results*. The study comprised 47 eyes. The mean follow-up time was 15.9 months (SD 12.2). The mean preoperative CDVA was 0.25 (SD 0.21). The final mean CDVA was 0.46 (SD 0.27). No hypotony or need for wound suturing was observed postoperatively. Mean postoperative refractive error was −0.27 Dsph (−3.87 Dsph to +2.85 Dsph; median 0.0, SD 1.28). The mean postoperative astigmatism was −1.82 Dcyl (min −0.25, max −5.5; median −1.25, SD 1.07). Postoperative complications were observed in 10 eyes. The most common complication was ovalization of the iris, which was observed in 8 eyes. The mean operation time was 35.9 min (min 11 min, max 79 min; median 34, SD 15.4).* Conclusion*. Retropupilary iris-claw intraocular lens (IOL) implantation with sutureless wound closing is an easy and fast method, ensuring good refractive outcome and a low risk of complication. The Haigis formula proved to be predictable in postoperative refraction.

## 1. Introduction

The development of intraocular surgical technique of refraction correction in aphakic eyes has been observed recently. Aphakia is commonly the result of complications arising from cataract surgery. The most common risk factors of intraoperative complication are weakness of zonular fibers mostly due to PEX or trauma. Despite a lack of capsular support or its insufficiency, when the implantation of intraocular lens (IOL) into the ciliary sulcus is unmanageable, it is still possible to achieve satisfactory refraction. There are many possibilities to provide acceptable refraction in such eyes by implanting IOL in the anterior or posterior segment of the eye during primary or secondary operation, which is still debatable. The location of the implantation and its method of fixation determine complexity of the surgery and potential side effects.

Placement of IOL in the anterior chamber (AC-IOL) is technically easy and fast but such location can harm corneal endothelium and structures of the anterior chamber angle. Growing evidence in the 1980s of complication connected with rigid closed-loop, angle-supported AC-IOL as endothelial cell loss leading to pseudophakic bullous keratopathy, uveitis, uveitis-glaucoma-hyphema syndrome, chronic macular edema, angle structure damage, formation of peripheral anterior synechiae, fibrosis of haptics into the angle, pupillary block, and hyphema led to the development of open-loop AC-IOLs; however they also induced complications [1]. For that reason they are contraindicated especially in patients with glaucoma or endothelial problems [2].

Sclera-fixated IOLs (SF-IOLs) are affordable and readily available. The IOL is located in natural position, near the focal point of the eye and further from corneal endothelium and structures of the angle. Different variants of sclera-fixation procedure are proposed, but they all are characterized by difficult intraocular manipulation and time-consuming surgery. Potential degradation of stitch and its interaction with sclera may be associated with suture erosion in the long term. Knot exposure may result in an increased incidence of endophthalmitis. Other possible complications include tilt and decentration of the IOL, open angle glaucoma, suprachoroidal hemorrhage, and retinal detachment [2, 3]. Although it was demonstrated that secondary SF-IOL implantation is associated with less early postoperative complications than primary AC- IOL, there were no long-term differences in the visual outcomes and complication profiles [3].

The iris-claw lens method was invented by Worst in 1980 in order to correct the refraction in aphakic eyes [4]. The principle of the lens fixation has remained unchanged for 30 years. As the decrease of endothelial cell density is observed [5], in order to avoid complications characteristic of the presence of an IOL in the anterior chamber, the technique of posterior fixation of iris-claw lenses was proposed by Amar [6] and later modified by Mohr et al. [7]. This technique preserves the natural anatomy of the eye. The popularization of this implantation technique has been observed recently. Although its implantation is technically easy, disadvantages of this method include the size of the incision, which when sutured usually generates astigmatism, and the relatively high cost of the IOL.

The aim of this study, therefore, was to analyze the results of sutureless iris-claw IOL retropupillary implantation during primary and secondary surgery.

## 2. Materials and Methods

The study of case series was comprised of consecutive patients operated on at the Ophthalmologic Clinic University Erlangen between March 2007 and May 2013 who underwent retropupillary implantation of Artisan/Verisyse iris-claw IOL (Ophtec BV; Advanced Medical Optics, Inc.). Data were collected retrospectively from hospital documentation for preoperative, intraoperative, and early postoperative period. Late postoperative follow-up data were collected with questionnaire from regional ophthalmological offices.

All patients were routinely fully informed about the risk and benefits of the surgery and the written consent was obtained.

Preoperative data included demographic data, corrected distance visual acuity (CDVA) measured with Snellen's decimal scale, refraction, intraocular pressure (IOP), preexisting pathology, history of the disease and former operations, and cause of the lack of the posterior capsule and biometry.

Intraoperative data included operation time, size and place of incision, course of operation, documentation of additional procedures, and intraoperative complications.

Postoperative data from follow-up visits included CDVA, refraction, and slit lamp findings, especially iris-related abnormalities. CDVA was measured with Snellen's chart and decimal notation. For CDVA analysis finger counting and hand movement were calculated as decimal values [8].

### 2.1. Biometry

Preoperative biometrical data from all patients were measured with optical method (IOL Master, Zeiss Meditec, Jena, Germany). The Haigis formula was used as calculation formula (ACD-Const: 4.21, A0-Const: −0,25, A1-Const: 0.4, and A2-Const: 0,1). For secondary implantation biometry performed prior to primary operation was used.

### 2.2. Refraction

Preoperatively, the refraction was measured with autorefractometry. The eyes which did not allow for autorefractometry due to cataract density were examined with subjective method. Corneal astigmatism was measured with IOL Master (Zeiss Meditec, Jena, Germany). Refraction was measured postoperatively with autorefractometer (RK-700 A; Nidek Co. Ltd., Gamagori, Japan). For this device deviation from the nominal value spherical and cylindrical vertex power is ±0.25 D for 0.00 to ±10.00 D and deviation from the nominal value of the ARK Cylinder axis for cylinder power is ±10° for 0.25 D to ±0.50 D, ±5° for >0.5 D to 3.00 D, and ±3° for >3.00 D.

### 2.3. Surgical Technique

All operations were performed by one experienced consultant. Because of the variety of cases and preexisting pathologies, the surgical procedures differed and were individually modified. All patients, however, had iris-claw IOL attached to the posterior surface of the iris. Anterior vitrectomy, posterior vitrectomy, removal of remnants of the capsule, and removal of IOL were performed if necessary. For IOL implantation a corneal or sclerocorneal tunnel was used. In most cases the existing cataract operation tunnel was extended to 5.5 mm. The IOL was implanted to the anterior chamber and moved with special tweezers through the iris to posterior chamber. With a help of the second instrument (spatula) haptics were attached to the iris in 3 and 9 o'clock position. No incision was stitched.

### 2.4. Statistical Analysis

For statistical analysis Kolmogorov-Smirnov test was applied to test for a normal distribution. Parametric *t*-test was used for comparison of variables (GraphPad Software Inc., La Jolla, USA). Differences were considered statistically significant at *p* < 0.05.

### 2.5. Patients

**Figure 1:**
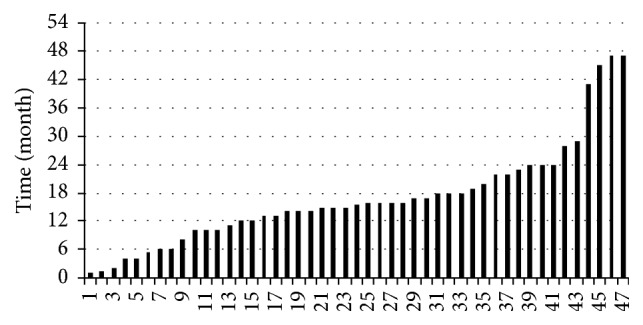
Observation time.

The study comprised 47 eyes (45 patients: 30 female and 15 male). The mean age of the patients was 73,6 years (range 35 to 91 years; median 78, SD 14.5). The mean follow-up time was 15.9 months (ranging from 1 to 47 month; median 13, SD = 12.2). Observation time is shown in Figure 1. Coexisting pathologies of the patients are shown in Table 1.

### 2.6. Indications

#### 2.6.1. Primary Operation

The iris-claw IOL was implanted during primary operation in 6 eyes (12.8%), in which local conditions did not allow for intracapsular or sulcus IOL implantation. In this group zonulysis occurred intraoperatively during complicated cataract surgery: in four eyes it was caused by PEX, in one eye by trauma, and in one eye by intraoperative floppy iris syndrome. In all these eyes anterior vitrectomy and capsule removal were performed. One eye additionally required pars plana vitrectomy due to luxated lens material.

#### 2.6.2. Secondary Operation

As a secondary operation iris-claw IOL was implanted in 41 eyes (87.2%) after previous cataract surgery: 24 eyes had subluxated and 3 eyes luxated posterior IOL; 14 eyes were aphakic after cataract surgery without IOL implantation was performed: 10 after complicated cataract operations and 4 after congenital cataract operations. In 2 cases lens material was luxated to the vitreous. In 15 eyes PEX was diagnosed; 3 eyes had trauma in medical history. The time period between cataract operation and iris-claw IOL implantation ranged from 1 day to 40 years.

### 2.7. Visual Acuity

The mean preoperative CDVA was 0.25 (ranging from hand movement to 1.0, median 0.2, SD 0.21). The final mean CDVA was 0.46 (ranging from hand movement to 1.0, median 0.4, SD 0.27). This improvement was significant (*p* < 0.0001, paired *t*-test). In 44.7% of the eyes final CDVA was equal to or higher than 0.5. CDVA was better than primary in 30 eyes (63.8%). In 7 eyes (14.9%) final CDVA was equal to initial CDVA. The deterioration of CDVA was found in 10 (21.1%) eyes. Six of them had PEX glaucoma; in one eye macular edema was observed prior to IOL implantation, one eye had macular atrophy in course of diabetic retinopathy, and two eyes were aphakic due to congenital cataract operation.

Changes of CDVA in all groups are shown in Table 2. In the group with glaucoma postoperative CDVA was significantly lower than in the rest of the groups (*p* = 0.017, unpaired *t*-test). Besides this no significant differences in preoperative and postoperative CDVA were observed in the remaining groups.

### 2.8. Refraction

**Figure 2:**
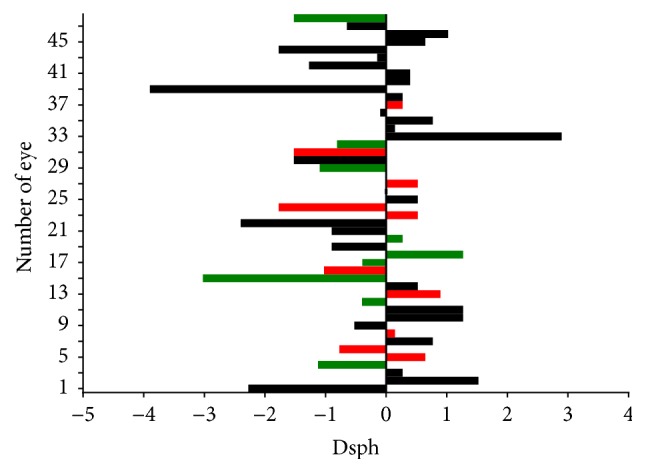
Refractive error. On the vertical axis: number of the eye. On the horizontal axis: refractive error in spherical equivalent. Green bars: eyes with iris/IOL complications and abnormalities; red bars: eyes with deceleration of CDVA.

The mean postoperative refractive error defined as the difference between target refraction and spherical equivalent of postoperative refraction was −0.27 Dsph (ranging from −3.87 Dsph to 2.85 Dsph; median 0.0, SD 1.28). It was within the range of ±1.0 Dsph in 61% of eyes. The achieved refraction data are distributed normally (Kolmogorov-Smirnov normality test *p* > 0.05). In the eyes with postoperative complications and abnormalities connected with iris and IOL mean refractive error was 0.10 Dsph (ranging from −1.31 Dsph to 1.34 Dsph; median −0.15, SD 0.71), which is not significantly different from the rest of the eyes (*p* = 0.75, unpaired *t*-test). The mean refractive error of all eyes is shown in Figure 2.

The mean postoperative astigmatism was −1.82 Dcyl (min −0.25, max −5.5; median −1.25, SD 1.07). Astigmatism increased in 79.1% and was reduced in 20.9% of eyes. Postoperatively it was lower than −1 Dcyl in 65,1% of cases. The mean difference between preoperative corneal astigmatism and postoperative total astigmatism was −0.30 Dcyl (maximal reduction was −3.39, maximal rise was −3.10, SD 1.26). The mean cylinder axis was preoperatively 81.7° (SD 48.8) and postoperatively 86.5° (SD 38.5). The mean shift of the axis was 30.41° (min 0°, max 89°; median 20°, SD 26.1).

**Figure 3:**
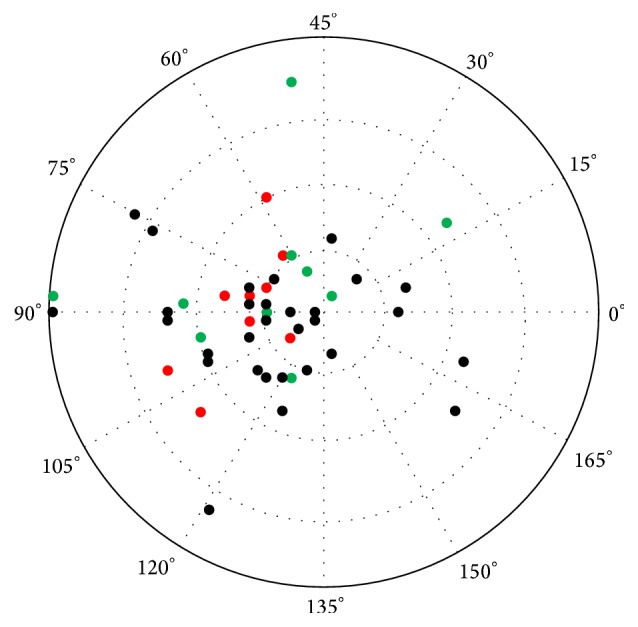
Postoperative astigmatism. Green dots: eyes with iris/IOL complications and abnormalities; red dots: eyes with deceleration of CDVA.

In the eyes with postoperative complications and abnormalities connected with iris and IOL, astigmatism was reduced in 40% and increased in 60% of eyes. The mean difference between preoperative corneal astigmatism and postoperative total astigmatism was −0.52 Dcyl (maximal reduction −1.45 Dcyl, maximal rise −2,9 Dcyl; SD 1.27). It was lower than −1 Dcyl in 40.0% of eyes. Mean shift of the cylinder axis was 38.25° (SD 32.7), which is not significantly different from the rest of the eyes (*p* = 0.32, unpaired *t*-test). The postoperative astigmatism of all eyes is shown in Figure 3.

### 2.9. Postoperative Abnormalities and Complications

During postoperative follow-up abnormalities were observed in 10 eyes (21.2%): 9 eyes with secondary and one with primary IOL implantation. They are shown in Table 3. The ovalization of the iris was the most common finding and it was observed in 8 eyes (17.0%). In 3 eyes iris atrophy (6.4%) occurred. No eye with iris atrophy had PEX. Only in one eye (2.1%) pigment dispersion was observed which did not result in IOP rise. In one eye, retinal detachment occurred, which was successfully treated with pars plana vitrectomy and gas tamponade. Previously this eye had IOL luxated to the vitreous. It was removed during pars plana vitrectomy with iris-claw IOL implantation. Only in one eye IOL decentration was observed. There was no significant difference in CDVA (*p* = 0.86, unpaired *t*-test) and IOP (*p* = 0.48, unpaired *t*-test) at the end of the observation period of the eyes with and without complications.

### 2.10. Intraocular Pressure

Intraocular pressure (IOP) was measured during every visit. In comparison to preoperative IOP (*p* = 0.06), postoperative IOP was not significantly changed at the end of the observation period. Postoperatively IOP was lower than 21 mmHg apart from one case with PEX glaucoma, where it reached 24 mmHg. In no case with postoperative iris/IOL complications was IOP elevation observed. At first postoperative day mean IOP was 14.8 mmHg which was not significantly (*p* = 0.15 paired *t*-test) lower than preoperatively with the lowest value of 8 mmHg. Despite the use of a sutureless technique, in no case was any sign of wound leakage, hypotony, or need for wound suturing was observed. Changes of IOP are shown in Table 4.

### 2.11. Operation Time

The operation time is shown in Table 5. The mean duration of operation was 35.9 min (SD 15.4). The shortest operation (11 min) was performed after complicated cataract surgery in aphakic eye, which did not require anterior or posterior vitrectomy. The most time-consuming operation (79 min) was performed in the eye with luxated IOL material. Eyes which required posterior vitrectomy took more operation time (which was not statistically significant).

## 3. Discussion

The best method of achieving acceptable refraction in eyes without capsular support for IOL is still a matter of discussion. Such conditions could appear after the lens or IOL luxation due to trauma or insufficient zonular fibers, for example, associated with PEX. In the following study PEX coexisted in 62% of the presented eyes. In most of the eyes it resulted in aphakia due to failed primary operation or to IOL luxation. Apart from trauma, PEX was also the cause of preoperative lens subluxation.

Retropupillary localization, due to increased distance from corneal endothelium and angle structures, has protective significance for endothelium and IOP rise, which is especially important for PEX and glaucoma patients. Both in this and in similar studies no clinical influence on corneal condition [9, 10] or intraocular pressure [9, 10] were observed, whereas after anterior fixation of iris-claw IOL IOP tended to rise in 9.5% of cases [11].

Implantation of iris-claw IOL onto anterior surface of the iris led to the reduction of endothelial cell density by 9.78% within 3 years [12] and up to 12.35% within 5 years [13], resulting in corneal decompensation in 1.7% within 2 years [11]. One of the possible explanations is intraocular manipulation in the anterior chamber [13]. It could also be attributed to the mechanical irritation of the anterior chamber due to IOL donesis [4]. The impact of mechanical manipulation in case of retropupillary IOL implantation should be even higher and donesis is the most frequent and obvious finding after iris-claw IOL implantation, which is not considered a complication.

The most common complication found in this study was ovalization of the iris. It had no influence on postoperative CDVA. A comparable frequency of ovalization of the iris was observed in other studies [9, 10, 14]. Ovalization, which could be explained by too tight enclavation in midperipheral iris stroma, tended to normalize over time [9]. Although biconvex architecture of the IOL and reduced contact with the iris surface should ensure no influence on stromal blood perfusion, one study indicated association of iris ovalization with the lack of iris perfusion associated with anterior implantation in phakic eye [15]. This factor could be related to iris atrophy, the second most frequent abnormality observed in this study. Atrophy of the iris is most common in places of enclavation and theoretically could be potentially associated with pigment dispersion [7]. Iris atrophy could also explain small tendency (up to 9% [9]) to decantation of the IOL.

PEX leads to degenerative and atrophic changes of the iris muscle cells [16]. Ovalization of the iris was observed in 3 eyes with PEX, suggesting that the significance of PEX in the explanation of this phenomenon is limited.

In the following study, the improvement of CDVA was achieved in 63% of the eyes. This result is similar to corresponding studies [10, 13]. Theoretically, noncomplicated IOL implantation should not influence CDVA. The observed deceleration rate of CDVA agrees with the results of similar studies [10]. It could be explained by progressive coexisting pathologies like PEX glaucoma and macular atrophy. The reduction of CDVA did not correlate with iris/IOL abnormalities which occurred in the observation time. Except for one eye diagnosed prior to the operation, no case of postoperative macular edema was reported. In other studies, macular edema after retropupillary IOL fixation is observed in 1.2% to 8.7% [7, 14]. The same frequency occurs in the case of anterior chamber iris-claw IOL [11]. It is comparable with the cases of AC-IOL and SF-IOL, where the rate of edema is observed in 2.7% to 10.4% [1, 17]. However, the limitation of this study is its retrospective character and the lack of the regular OCT screening.

The iris-claw IOL implantation rarely correlates with retinal detachment [11, 14]. It is difficult to determine, if it had any association with the IOL or the operation technique. In one particular case in this study the eye had former vitrectomy due to IOL luxation to the vitreous. It should be remembered that most of the eyes which required secondary IOL implantation had pathologies which may cause retinal detachment. In the cases of scleral fixation retinal detachment is observed more frequently and suprachoroidal hemorrhage could occur [3], which was not observed in iris fixation. In the case of sclera fixation it could be explained by major intraoperative mechanical manipulation in posterior segment.

Most manipulations during retropupillary iris fixation are performed in the anterior chamber where haptics are more controllable and can be easily observed. Even then retropupillary iris-claw IOL implantation is quite an easy technique, resulting in twice as short an operating time and significantly shorter time in aphakic cases in comparison to sclera fixation. Even during primary complicated cataract surgery combined with posterior vitrectomy the mean operation time was shorter than it was reported in cases of scleral fixation in aphakic eyes [16].

In this study the Haigis formula for IOL power calculation was used providing −0.27 ± 1.28 D of the mean postoperative refraction error. Other authors used the SRK II formula with A-constant of 116.8 [10] or the SRK/T formula with constant of 116.9 [9], which resulted in 0.43 ± 1.93 D and 0.00 ± 1.21 D of refractive error, respectively. The SRK/T formula with A-constant of 116.5 resulted in −1.42 D ± 1.22 D in posttraumatic and −1.5 ± 1.15 in postcataract surgery aphakic group [14]. In the case of anterior chamber implantation A-constant of 115.0 was used resulting in +0.12 ± 1.76 D [11]. Although the Haigis formula in this study has better postoperative refractive results compared to the other formulas [18], it requires anterior chamber depth defined as the distance from the corneal vertex to the anterior lens capsule which is not possible in aphakic eyes. Therefore, it could be used only in cases with biometry performed before primary operation.

Verisyse IOL has rigid PMMA construction; it requires large, at least 5.5 mm incision, which is likely to induce a high amount of surgery induced astigmatism. In this study the mean difference between postoperative and preoperative corneal astigmatism was −0.86 Dcyl. In 72.8% it was less than 1 Dcyl, which is even less than that in cases with implantation of such IOL through scleral tunnel incision, where it reached −2.01 Dcyl [10]. Closing the wound with the Nylon 10-0 suture with the use of the same implantation technique generated slightly higher (−3.64 ± 3.34 Dcyl) astigmatism, suggesting that the suture played a moderate role in deformation of corneal surface [10]. To reduce postoperative astigmatism different incision site and design can be used alternatively with combination of corneal refractive surgery techniques such as limbal relaxing incisions, LASIK, or PRK [19]. In presented study in the most cases the incision architecture was determined by primary tunnel incision localization. The problem of postoperative astigmatism could probably be reduced with foldable lenses, which can be inserted through 3 mm incisions.

Another possible problem with such a large, nonsutured incision could be leakage and hypotonia. Although no wound in this study was closed with sutures, no signs of leakage, bleb formation, or hypotony were observed postoperatively.

Due to retrospective character of this study, limitations of this study are lack of statistical power analysis, small subgroup sample size, and various observation time. This is, however, to the best of our knowledge the first analysis of the results of sutureless iris-claw IOL retropupillary implantation during primary and secondary surgery.

## 4. Conclusions

Retropupillary iris-claw IOL combines the ease of anterior chamber IOL implantation with optical and physiological advantages of posterior IOL location, ensuring a good refractive outcome and a low risk of complication. With careful wound construction surgery does not require suturing, which can reduce generated astigmatism. To our knowledge, the application of the Haigis formula in retropupillary iris-claw IOL was for the first time reported in postoperative refraction calculation.

This type of implantation should be considered especially in all aphakic patients with contraindications for anterior chamber implant because of glaucoma or endothelial abnormality. The most common abnormalities after retropupillary iris-claw IOL implantation are ovalization and atrophy of the iris, which have no influence on visual or refractive outcomes as well as on intraocular pressure. The same concerns patients with glaucoma and PEX. Retropupillary iris-claw IOL implantation is a safe and relatively fast method in the cases of iatrogenic failure, which does not allow for intracapsular or sulcus implantation during primary complicated cataract surgery.

## Figures and Tables

**Table 1:** Coexisting preoperative ocular pathologies.

Pathology	Eyes number
PEX syndrome	19
PEX glaucoma	10
Glaucoma	7
St p trabeculectomy	5
St p trauma	4
Diabetic retinopathy	2
St p congenital cataract	2
St p pars plana vitrectomy	2
Macular oedema	2
St p CFC	3
Myopia magna	1
St p anterior uveitis	1
Epiretinal membrane	1

**Table 2:** CDVA change.

	*N*	Preoperative CDVA			Final CDVA		
		Mean	Median	SD	Mean	Median	SD
All	47	0.25	0.2	0.21	0.46	0.4	0.27
Primary implantation	6	0.21	0.2	0.16	0.52	0.5	0.20
Secondary implantation	41	0.35	0.2	0.62	0.45	0.4	0.28
Aphakic	14	0.56	0.3	1.03	0.60	0.7	0.36
Dislocated IOL	27	0.25	0.2	0.19	0.39	0.3	0.22
PEX syndrome	19	0.22	0.2	0.14	0.37	0.3	0.23
Glaucoma	17	0.28	0.3	0.19	0.34	0.2	0.20
Post-op abnormalities	10	0.28	0.3	0.18	0.51	0.5	0.23

**Table 3:** Postoperative complications and abnormalities.

	*N*	%
*All *	10	22.2
Oval iris	8	17
Atrophy of iris	3	6.4
IOL decentration	1	2.1
Retinal detachment	1	2.1

**Table 4:** Changes in intraocular pressure before and after operation.

	Preoperative			Final		
	Mean	Median	SD	Mean	Median	SD
All	16.7	15	5.4	15.4	15	3.4
Glaucoma	21.0	17.5	9.9	13	13	4.1
PEX glaucoma	19.4	19	6.8	15.9	14.5	5.6
PEX syndrome	16.0	15.5	4.3	15.1	15	1.2
Complications	16.2	14.5	7.2	14.6	14.5	1.2

**Table 5:** Operation time.

	Mean	Median	SD	Min	Max
All	35.9	34	15.4	11	79
Primary implantation	38.4	34	18.4	22	67
Secondary implantation	35.6	34	15.3	11	79
With additional posterior vitrectomy	44.2	37	18.0	16	79
